# A308 AN UNUSUAL CASE OF MACE DYSFUNCTION: THE BENEFIT OF USING ANTEGRADE CONTRAST FOLLOW THROUGH MIMICKING HOME SETTINGS

**DOI:** 10.1093/jcag/gwad061.308

**Published:** 2024-02-14

**Authors:** D Avelar Rodriguez, J Langer, M Gould

**Affiliations:** The Hospital for Sick Children, Toronto, ON, Canada; The Hospital for Sick Children, Toronto, ON, Canada; The Hospital for Sick Children, Toronto, ON, Canada

## Abstract

**Background:**

Malone Antegrade Continence Enema (MACE) is indicated in cases where medical management for constipation or faecal incontinence with laxatives or enemas has failed. MACE allows the administration of flushes with and without laxatives in an antegrade manner to mechanically empty the colon.

**Aims:**

The aim of this case report is to illustrate an unusual case of MACE dysfunction and how it was troubleshooted.

**Methods:**

Case report and review of the literature

**Results:**

11-year-old female with neurogenic constipation and faecal incontinence for which MACE was being used.

She was referred to clinic for evaluation of new-onset vomiting, abdominal distention, and abdominal pain with MACE flushes. Her flushes consisted of crushed Bisacodyl, glycerin, PEG 3350 and 300 ml of saline solution. Cone enemas were also being used to completely empty her rectum, otherwise evacuation with regular flushes was not occurring, but was causing significant abdominal pain, distention and vomiting.

A contrast study was done by flushing water-soluble contrast through the MACE in an antegrade manner, while simultaneously performing a contrast enema, demonstrating good passage of contrast with no findings suggestive of obstruction.

She was admitted to hospital to complete imaging studies, bowel clean out and to better understand her MACE dysfunction. Following bowel clean out in hospital, MACE flushes were resumed, causing the same symptoms. Flushes without Bisacodyl were tried in the attempt to avoid possible colonic spasms; however symptoms re-occurred.

A second contrast study was performed (figure 1), mimicking home flushes in volume and time of administration, as well as patient’s position (sitting), completed in an antegrade fashion only. The patient developed symptoms 20 minutes after administering the contrast requiring cone enema. By the end of the study, there was poor passage of contrast through the proximal descending colon, suggesting a kink at the juncture between the sigmoid and descending colon. There was no reflux on contrast into the terminal ileum. An x-ray was done around 5 hours after, and contrast was still not seen in the rectum.

The patient underwent diagnostic laparoscopy, finding adhesions in the left upper quadrant of the descending and sigmoid colon, causing a Z-configuration hairpin obstruction, and colostomy creation. The patient has been doing well since colostomy creation with no pain.

**Conclusions:**

MACE dysfunction can be caused by colonic adhesions distal to the MACE site Simultaneous antegrade contrast through MACE and contrast enema may miss bowel obstruction Antegrade contrast study mimicking home settings may be more helpful in situations where bowel obstruction is suspected

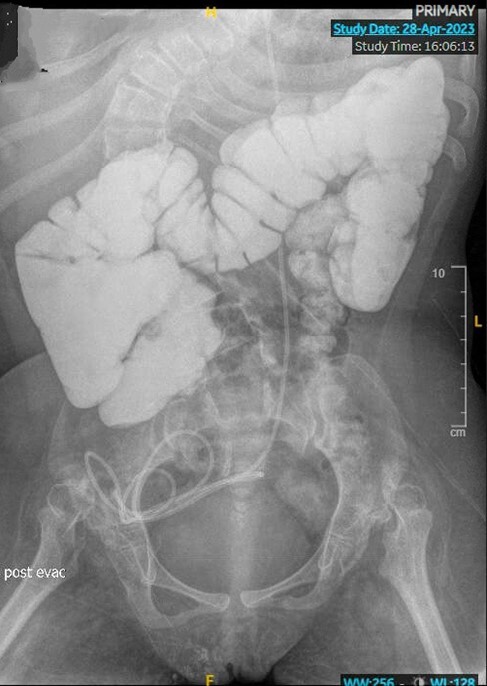

**Funding Agencies:**

None

